# Supplementary material for: Mapping the evolution of fertility support policies in China: A content and instrumental analysis
Source: PLoS One. 2025 Oct 9;20(10):e0332137. doi: 10.1371/journal.pone.0332137 (PMC12510515; doi:10.1371/journal.pone.0332137)
Supplement: S1 Appendix — (ZIP) [file pone.0332137.s001.zip › S1 Appendix. 226 original policy documents/129-卫生部关于省级妇幼保健院办院方向的若干意见(FBM-CLI-4-32910).doc]

卫生部关于省级妇幼保健院办院方向的若干意见

**发布部门：** [卫生部(已撤销)](https://www.pkulaw.com/law/chl?Aggs.IssueDepartment=60222) [机构沿革](javascript:void(0))

**发布日期：**1990.09.30

**实施日期：**1990.09.30

**时效性：** [现行有效](https://www.pkulaw.com/law/chl?Aggs.TimelinessDic=01)

**效力级别：** [部门规范性文件](https://www.pkulaw.com/law/chl?Aggs.EffectivenessDic=XE0302)

**法规类别：** [医疗保健](https://www.pkulaw.com/law/chl?Aggs.Category=09623)

卫生部关于省级妇幼保健院办院方向的若干意见
（１９９０年９月３０日）

　　妇幼卫生工作是我国卫生工作的重要组成部分，在实现“２０００年人人享有卫生保健”的战略目标、落实计划生育基本国策、开展优生优育工作中担负着日益繁重的任务。省级妇幼保健机构作为全省妇幼卫生工作的业务指导中心，在推动我国妇幼卫生事业的发展中至关重要。针对当前省级妇幼保健机构尚不健全、办院方向不够明确，保健力量薄弱、隶属关系不一等问题，为加强治理整顿、把握办院方向、强化保健职能、明确工作范围、理顺隶属关系，现对省级妇幼保健院的办院方向提出以下几点意见，请各地认真遵照执行。
　　一、名称
　　省级妇幼保健机构，一律称为“××省（市、自治区）妇幼保健院”。

　　二、级别与管理
　　省级妇幼保健院的级别与同级医疗、防疫机构相同（即省人民医院、省防疫站为正处级的，省妇幼保健院也为正处级；省人民医院、省防疫站为副厅级的，省妇幼保健院也为副厅级）。省级妇幼保健院为事业单位，直属省、自治区、直辖市卫生厅（局）领导，由妇幼处管理。

　　三、办院方针
　　以《[妇幼卫生工作条例](https://www.pkulaw.com/chl/bc5930fa7a83fc0cbdfb.html)》（卫生部〔８６〕卫妇字５号）文件为办院依据，坚持以保健为中心，指导基层为重点，保健与临床相结合的办院方针。

　　四、主要职能与工作范围
　　（一）保健工作
　　１．掌握本省孕产妇死亡率、婴儿死亡率等变化情况，针对主要死因提出干预策略，协助卫生行政部门制定干预规划并参与实施；
　　２．掌握本省影响儿童生长发育、影响儿童与妇女身体与心理健康的主要生物、社会、环境与心理因素，针对这些主要因素提出预防措施；协助卫生行政部门制定规划并参与实施；
　　３．为地（市）级妇幼保健机构提供技术指导与技术支持；
　　４．负责全省妇幼卫生业务工作的质量监测和质量审评，并定期向卫生行政部门提交报告；
　　５．协助卫生行政部门管理妇产医院、儿童医院和综合医院中的妇幼保健业务；
　　６．开展妇女保健、儿童保健、计划生育技术服务、优生保健和妇女、儿童常见病防治工作。
　　（二）临床工作
　　１．接受下级妇幼保健机构的转诊；
　　２．处理妇女、儿童高危病例；
　　３．开展与妇女、儿童保健目标一致的其它临床服务；
　　４．提供与妇女、儿童保健密切有关的检测技术。
　　（三）培训工作
　　１．根据本省妇幼保健专业技术人员配置情况，协助卫生行政部门制定人力发展规划与在职培训计划；
　　２．接受县和县级以上妇幼保健机构专业技术人员进修、培训和妇幼卫生专业学生实习；
　　３．协助卫生行政部门对妇幼保健专业技术人员进行业务考核。
　　（四）健康教育
　　１．根据妇女、儿童保健目标，协助卫生行政部门制定健康教育计划，指导下级妇幼保健机构的健康教育活动；
　　２．提供健康教育材料；
　　３．开展群众性的健康教育。
　　（五）妇幼卫生信息工作
　　１．根据卫生部提出的信息指标，收集、整理、分析、储存全省妇幼保健信息资料；
　　２．将经过整理分析的信息资料，按规定时间上报省和国家卫生行政部门；同时反馈给地（市）级妇幼保健机构；
　　３．指导下级妇幼保健机构的信息工作，抽查、核实信息资料。
　　（六）科研工作
　　１．针对影响妇女、儿童身体和心理健康的主要问题，开展应用性科研活动；
　　２．推广妇女保健、儿童保健、计划生育服务和优生优育适宜技术；
　　３．承担国家或省下达的科研任务；
　　４．指导地、市、县级妇幼保健机构的科研活动并提供技术支持。

　　五、内设机构
　　省级妇幼保健院实行院、部（副处级）两级行政管理体制。院内设保健部和临床部。各部依据所担负的职能与工作范围，设置相应的业务科（室）。科（室）设置应体现保健为主，面向群体、面向基层和精简高效的原则，由省、市、自治区卫生厅（局）审查批准，并报卫生部妇幼卫生司备案。

　　六、编制标准
　　（一）按照卫生部、劳动人事部（８６）卫妇字第２号文件颁发的《[各级妇幼保健机构编制标准（试行）](https://www.pkulaw.com/chl/72eb199a7b6e35dcbdfb.html)》执行。
　　（二）“八·五”期间按照二类编制标准，省级妇幼保健院保健部应为８０—１２０人，最低不得少于８０人，其中卫生技术人员应占７５—８０％；临床部人员按设立的床位数，依１∶１．７增加编制，增加的编制中卫生技术人员亦应占７５—８０％。
　　（三）临床部设置床位数，应根据实际需要和地方政府“区域卫生规划”的原则确定。一般应设５０—２００张病床。

　　七 、专业人员轮换制度
　　省级妇幼保健院中的住院医师在保健部和临床部之间定期轮换，一般每年轮换一次，特殊情况下在同一部门不得超过两年。临床部主治医师在晋升副主任医师之前，必须在保健部工作一年；保健部主治医师在晋升副主任医师之前，必须在临床部工作一年。
　　八、装备标准
　　省级妇幼保健院装备标准，根据卫生部（８８）卫妇字第３号颁发的《妇幼保健机构各级管理人员和技术人员职责》等３个文件的通知精神，按《妇幼保健机构仪器设备标准》中“省、市级妇幼保健单位仪器设备标准”执行。

　　九、预算管理形式
　　省级妇幼保健院实行全额预算管理形式。

　　十、考核指标
　　卫生部妇幼司将依据省级妇幼保健院的职能要求和工作范围、本文所列各项要求及国务院与有关部委的文件精神，另行制定省级妇幼保健院的分类考核指标。关于妇幼保健机构的分级管理标准由卫生部另行规定。
　　各省、自治区、直辖市卫生行政部门，应在现有基础上，对省级妇幼保健机构进行治理整顿，逐步充实完善，提出经过论证的调整计划，并将任务具体落实为年度计划，以保证在“八·五”期末完成调整任务，建设成与其基本职能、当地妇女、儿童保健需求以及社会经济发展水平相适应的省级妇幼保健院。
　　尚未建立省级妇幼保健院的省（市），可按（８６）卫妇字第２号《关于[各级妇幼保健机构编制标准（试行）的通知](https://www.pkulaw.com/chl/72eb199a7b6e35dcbdfb.html)》、（８６）卫妇字第５号《[妇幼卫生工作条例的通知](https://www.pkulaw.com/chl/bc5930fa7a83fc0cbdfb.html)》及本《意见》精神，力争“八·五”期间纳入地方发展规划，完成组建工作。
c27080--010116wwj

© 北大法宝：（[www.pkulaw.com](https://www.pkulaw.com/)) 专业提供法律信息、法学知识和法律软件领域各类解决方案。北大法宝为您提供丰富的参考资料，正式引用法规条文时请与标准文本核对。
欢迎查看所有[产品和服务](https://www.pkulaw.com/helps/2.html)。法宝快讯：[如何快速找到您需要的检索结果？](https://www.pkulaw.com/helps/70.html)[法宝](https://www.pkulaw.com/helps/69.html)V6有何新特色？

扫描二维码阅读原文

**原文链接：**<https://www.pkulaw.com/chl/5db8749d4955a0e9bdfb.html>
